# Targeted nanoparticles encapsulating (−)-epigallocatechin-3-gallate for prostate cancer prevention and therapy

**DOI:** 10.1038/srep41573

**Published:** 2017-02-01

**Authors:** Vanna Sanna, Chandra K. Singh, Rahime Jashari, Vaqar M. Adhami, Jean Christopher Chamcheu, Islam Rady, Mario Sechi, Hasan Mukhtar, Imtiaz A. Siddiqui

**Affiliations:** 1 Department of Chemistry and Pharmacy, Laboratory of Nanomedicine, University of Sassari, 07100 Sassari, Italy; 2 School of Medicine and Public Health, Department of Dermatology, University of Wisconsin-Madison, Madison WI 53706, USA; 3 Department of Zoology, University of AL-Azhar, Cairo, Egypt

## Abstract

Earlier we introduced the concept of ‘*nanochemoprevention*’ i.e. the use of nanotechnology to improve the outcome of cancer chemoprevention. Here, we extended our work and developed polymeric EGCG-encapsulated nanoparticles (NPs) targeted with small molecular entities, able to bind to prostate specific membrane antigen (PSMA), a transmembrane protein that is overexpressed in prostate cancer (PCa), and evaluated their efficacy in preclinical studies. First, we performed a molecular recognition of DCL- and AG-PEGylation on ligand binding on PSMA active site. Next, the biocompatible polymers PLGA-PEG-A were synthesized and used as base to conjugate DCL or AG to obtain the respective copolymers, needed for the preparation of targeted NPs. The resulting EGCG encapsulating NPs led to an enhanced anti-proliferative activity in PCa cell lines compared to the free EGCG. The behavior of EGCG encapsulated in NPs in modulating apoptosis and cell-cycle, was also determined. Then, *in vivo* experiments, in mouse xenograft model of prostatic tumor, using EGCG-loaded NPs, with a model of targeted nanosystems, were conducted. The obtained data supported our hypothesis of target-specific enhanced bioavailability and limited unwanted toxicity, thus leading to a significant potential for probable clinical outcome.

We and others have repeatedly verified the efficacy of green tea polyphenol, epigallocathechin-3-gallate (EGCG), in various preclinical models of human cancer(s) including that of the prostate gland, however the applicability of its use to human has met with only limited success, largely due to inefficient systemic delivery and bioavailability^1^,^2^,^3^,^4^,^5^,^6^,^7^,^8^,^9^,^10^,^11^,^12^,^13^,^14^,^15^. We envisioned that nanoparticle-mediated delivery could be useful to control the toxicity and enhance the bioavailability of the chemopreventive agents, and introduced the concept of “*nanochemoprevention*”^16^,^17^,^18^. Our studies demonstrated that EGCG encapsulated in polymeric nanoparticles (NPs) exhibited over ten-fold dose advantage for exerting its pro-apoptotic and anti-angiogenic effects against prostate cancer (PCa), both *in vitro* and *in vivo*^16^. In addition, we later prepared and verified the efficacy of a different EGCG nanoformulation in PCa model systems^19^,^20^,^21^.

On the other hand, active targeting via the inclusion of a specific ligand on the NPs is expected to provide a powerful strategy for the development of effective anticancer therapeutics^22^,^23^,^24^,^25^,^26^,^27^. Overexpressed tumor-associated membrane receptors appeared to be suitable targets for pharmacological intervention^28^. For PCa treatment, prostate-specific membrane antigen (PSMA), a well-known transmembrane protein that is overexpressed on PCa epithelial cells, has demonstrated a promising potential for the management of PCa^29^,^30^,^31^. To this end, functionalization of NPs with ligands that bind the extracellular domain of these receptors can be suited for selective delivery of bioactive molecules to diseased cells^32^,^33^,^34^,^35^,^36^.

There have been intense efforts to develop targeted drug delivery vehicles for successful therapy of several human cancers including the cancer of prostate gland^37^,^38^,^39^,^40^,^41^,^42^. Studies have verified the use of antibodies, aptamers, peptides, and small molecules for the organ specific targeting of therapeutic agents^22^,^23^,^24^,^25^,^26^,^27^,^32^,^33^,^34^,^35^,^36^.

In this study, we planned to develop novel targeted polymeric EGCG-loaded biocompatible NPs, coated on their shell surface by small organic molecules as targeting ligands with specificity for PSMA. The goal of this study were to (i) develop novel targeted EGCG-loaded NPs to achieve active cellular targeting, (ii) investigate their anti-proliferative and mechanistic activity as compared to the non-targeted NPs and native EGCG, and, (iii) evaluate the *in vivo* preclinical efficacy of EGCG-encapsulated nanosystems. Specifically, we obtained three types of polymeric NPs (Fig. 1), constituted by non-targeted NPs (i.e., A-EGCG NPs), and NPs targeted with either the pseudomimetic dipeptide DCL (i.e., DCL-EGCG NPs), or with Asp-Glu – AG (i.e., AG-EGCG NPs). First, to explore the interaction of DCL- and AG-PEGylation with the PSMA, a comparative computational docking study was performed. Next, NPs were prepared by nanoprecipitation method and were fully characterized in terms of morphology and physicochemical properties, as well as for EGCG-content and EGCG-release. The anti-proliferative effects of our nanoformulations were investigated on LNCaP (hormone-sensitive cells), and PC-3 and DU-145 (hormone-independent line possessing dysfunctional androgen receptors) PCa cell lines, compared to those of free EGCG. Moreover, the cellular uptake of the nanosystems was explored by fluorescence microscopy. Furthermore, gel electrophoresis analyses and flow cytometry were conducted to evaluate the induction of apoptosis and modulation of death receptor pathway, and to elucidate the impact of nanoencapsulation on the mechanism of action. Finally, the effects of EGCG and its nanoformulations were studied *in vivo* using athymic nude mice xenograft model. In addition, tumor growth inhibition was also monitored by quantitative determination of prostate specific antigen (PSA) levels in serum.

## Materials and Methods

### Molecular Modeling Studies

Ligand docking studies were performed using Molecular Operating Environment (MOE) 2009.10^43^. Three-dimensional structure of PSMA (GCPII) protein complexed with GPI-18431 inhibitor used for docking experiments was retrieved from the PDB (Protein Data Bank; http://www.rcsb.org/ - PDB code: 2C6C^44^). Before docking, the protein structure was properly protonated using the Protonate 3D option, and was geometrically optimized and minimized by employing AMBER99 force field with a generalized Born solvation model. The partial charges were automatically calculated. The structures of DCL, AG, as well as the model polymers PEG-DCL, were built in MOE and minimized before the docking, using the MMFF94x force field, with the systematic algorithms until a RMSD gradient of 0.001 kcal mol^−1^ Å^−1^ was reached. The protonation states of amine and carboxy moieties of ligand structures were set properly, and rotatable bonds were kept flexible throughout the docking procedure. Rigid receptor-flexible ligand docking calculation were performed using the docking simulation feature MOE-dock by setting grid sizes that included the entire macromolecule. Only the best scored poses generated in the docking experiments were retained and examined with MOE.

### Chemistry

Poly (D,L-lactide-co-glycolide) carboxylic acid end group, (PLGA-A, Purasorb Polymer PDLG 5002 A, Mw ~17,000) (lactide/glycolide ratio of 50:50, inherent viscosity: 0.20 dL/g in CHCl_3_) was kindly provided by Corbion Purac (Gorinchem, The Netherlands). The heterofunctional PEG polymer with a terminal amine and carboxylic acid functional group, NH_2_-PEG–COOH (MW 3400), was purchased from JenKem Technology USA. Poly-(ε-caprolactone) (PCL, Mw ~80,000), Pluronic^®^ F-127 was obtained from Sigma-Aldrich (Steinheim, Germany). (−)-Epigallocatechin gallate (EGCG) (98%), was supplied by Zhejiang Yixin Pharmaceutical Co., Ltd. (Lanxi, Zhejiang, China), while, L-Aspartyl-L-Glutamate (AG) dipeptide (Asp-Glu ≥95%) and all solvents and other chemicals (used for the preparation of DCL and *pseudo*-tri-block-copolymers) were purchased from Sigma-Aldrich, Carlo Erba or ORPEGEN Peptide Chemicals GmbH. All reagents of commercial quality were used without further purification. Melting points (mp) were determined using an Electrothermal melting point or a Köfler apparatus and are uncorrected. Nuclear magnetic resonance (^1^H-NMR) spectra were determined in CDCl_3_, DMSO-d_6_ or CDCl_3_/DMSO-d_6_ (in 3/1 ratio) and were recorded at 400 MHz on a a Bruker Avance III. Chemical shifts are reported in parts per million (ppm) downfield from tetramethylsilane (TMS), used as an internal standard. Splitting patterns are designated as follows: s, singlet; d, doublet; t, triplet; q, quadruplet; m, multiplet; brs, broad singlet; dd, double doublet. The assignment of exchangeable protons (OH and NH) was confirmed by the addition of D_2_O. Electron ionization and MALDI TOF mass spectra (70 eV) were recorded on a Hewlett-Packard 5989 Mass Engine Spectrometer and by a MALDI micro MX (Waters, micromass) equipped with a reflectron analyser. Mass calibration was carried out by using the antocyan mixture provided by the manufacturer as a standard. UV-vis absorption spectra were registered by a spectrophotometer uniSPEC 2 (LLG Labware, BDL Czech Republic sro, Turnov, Czech Republic). Analytical thin-layer chromatography (TLC) was carried out on Merck silica gel F-254 plates. Flash chromatography purifications were performed on Merck Silica gel 60 (230–400 mesh ASTM) as a stationary phase. The purity of copolymers was determined by high performance liquid chromatography (HPLC) using an HP 1200 (Agilent Technologies, USA) system, equipped with a Hypersil BDS C18 column (Alltech Italy, 250 × 4.6 mm i.d., 5 μm particle size); these materials were found to be >95% pure. Elemental analyses for DCL and its intermediates were performed on a Perkin-Elmer 2400 spectrometer at Laboratorio di Microanalisi, Dipartimento di Chimica, Università di Sassari (Italy), and were within ±0.4% of the theoretical values.

### Synthesis of PLGA-PEG-A Block Copolymer

To a solution of PLGA–COOH (5.0 g, 0.28 mmol) in anhydrous methylene chloride (25 mL), N-hydroxysuccinimide (NHS, 127 mg, 1.1 mmol, ~4 equiv) and 1-ethyl-3-(3-dimethylaminopropyl)-carbodiimide (EDC, 233 mg, 1.2 mmol, ~4.3 equiv.) were added, and the reaction mixture was magnetically stirred at room temperature for 12 h under nitrogen atmosphere. PLGA–NHS was obtained by precipitation with cold diethyl ether (15–20 mL) as a white solid, which was filtered, and repeatedly washed in a cold mixture of diethyl ether and methanol (few drops) to remove residual NHS, then dried with nitrogen and put under vacuum to remove solvent (yield: ~98%). The activated intermediate PLGA–NHS (5.0 g, 0.28 mmol) was dissolved in anhydrous chloroform (15–20 mL), NH_2_–PEG–COOH (1.25 g, 0.37 mmol, 1.3 equiv) and N,N-diisopropylethylamine (DIPEA) (140 mg, 1.08 mmol, 3.8 equiv) were then added under magnetic stirring, thus the reaction mixture was magnetically stirred at room temperature for 24 h. The desired copolymer was precipitated with cold diethyl ether, and treated with the same solvent to remove unreacted PEG as described above (yield: ~95%). The resulting PLGA–PEG block copolymer was dried under vacuum, characterized by ^1^H-NMR (400 MHz), and used for NPs preparation without any further purification. ^1^H-NMR (400 MHz, CDCl_3_) δ 5.23 (m, OC-C*H*(CH_3_)O, PLGA), 4.78 (m, OC-C*H*_2_O, PLGA), 3.65 (s, C*H*_2_C*H*_2_O, PEG), 1.56 (brs, OC-CHC*H*_3_O, PLGA)^36^.

### Preparation of 2-{[(5-amino-1-carboxypentyl)carbamoyl]amino}pentanedioic acid (DCL)

The ligand DCL was synthesized according with our previously reported procedure^36^. The intermediate (S)-2-[3-(5-amino-1-tert-butoxycarbonylpentyl) ureido]pentanedioic acid di-tert-butyl ester **1**, (500 mg, 0.65 mmol) was dissolved in 15 mL of 1:1 trifluoroacetic acid (TFA):methylene chloride (CH_2_Cl_2_) solution and stirred at room temperature for 4 h. Then, the solution was evaporated under reduced pressure. The solid residue was triturated with dry diethyl ether, filtered, and washed three times (with the same solvent) to yield DCL as a beige powder (yield: ~92%). ^1^H-NMR (400 MHz, DMSO-d6) δ 7.80–7.60 (brs, 3 H), 6.40–6.30 (s, 2 H), 4.15–4.00 (m, 2 H), 3.78–3.55 (m, 2 H), 3.46–3.20 (brs, 1 H), 2.78–2.72 (d, 2 H), 2.27–2.22 (m, 2 H), 2.00–1.85 (m, 2 H), 1.76–1.59 (m, 2 H), 1.53–1.47 (m, 2 H), 1.38–1.19 (m, 2 H). MS (MALDI-TOF): [M^+^ 319]; EI: [M^+^ 319]. Anal. (C_12_H_21_N_3_O_7_ • 1.0CF_3_COOH) C, H, N.

### Preparation of 2-{3-[1-p-Methoxybenzylcarboxylate-(5-tert-butylcarbamylpentyl)]-ureido}-di-p-methoxybenzyl pentanedioate (1)

Triphosgene (0.66 g, 2.26 mmol) was placed to a flamedried flask cooled to 0 °C, under nitrogen atmosphere, and a solution of **3**^36^ (2.5 g, 6.82 mmol, 3 equiv) and TEA (1.94 mL, 13.63 mmol, 6 equiv) dissolved in CH_2_Cl_2_ (10 mL) was added. The reaction mixture was stirred at 0 °C for 20 min. A mixture of **2**^36^ (2.90 g, 6.82 mmol, 3 equiv) and TEA (1.94 mL, 13.63 mmol, 6 equiv) in CH_2_Cl_2_ (10 mL) was then added, the resulting mixture was allowed to warm to room temperature and stirred for 2 h. Product was extracted by CH_2_Cl_2_, washed with water and brine, and dried over Na_2_SO_4_. Purification by flash chromatography (20/80 EtOAc/CH_2_Cl_2_) afforded a yellow oil that solidified upon standing (yield: ~73%). ^1^H-NMR (400 MHz, CDCl3) δ: 7.26 (d, 6 H), 6.87 (d, 6 H), 5.52 (d, 2 H), 5.13–4.98 (m, 6 H), 4.76 (brs, 1 H), 4.53–4.48 (m, 1 H), 4.50–4.43 (m, 1 H), 3.79 (s, 9 H), 3.04–2.96 (m, 2 H), 2.40–2.34 (m, 2 H), 2.14–2.11 (m, 2 H), 1.94–1.70 (m, 2 H), 1.60–1.56 (m, 2 H), 1.42 (s, 9 H), 1.25–1.22 (m, 2 H). EI: 780 [M + 1]^+^. Anal. (Calculated for C_41_H_53_N_3_O_12_) C, H, N.

### Preparation of bis-4-methoxybenzylglutamate hydrochloride (2) and 2-amino-6-tert-butoxycarbonylamino-hexanoic acid 4-methoxybenzyl ester (3)

The intermediates **2** and **3** were re-prepared according to our previously published procedures^36^, starting from a large amount of starting materials (i.e., calculated considering a total amount of ~20 g of Nε-Boc-N-Fmoc-L-lysine, as base for the preparation of **3**). Characterization for these compounds was coherent with that reported in literature.

### Synthesis of PLGA-PEG-DCL and PLGA-PEG-AG *pseudo*-tri-Block Copolymers

Conjugation of targeting agent DCL or AG to functional PEG terminus was performed with the use of electrophilic NHS activated esters (i.e., PLGA-PEG-NHS) of PEG carboxylic acids. Reaction between terminal amine group of DCL or AG and PLGA-PEG-NHS resulted in the formation of chemically stable amide bonds. To a solution of PLGA–PEG-COOH di-block copolymer (3.0 g, 0.1391 mmol) in anhydrous methylene chloride (10 mL), NHS (80 mg, 0.69 mmol, 5 equiv) and EDC (160 mg, 0.83 mmol, 6 equiv) were added, and the solution was magnetically stirred at room temperature for 12 h, under nitrogen atmosphere. The activated PLGA–PEG-NHS copolymer was precipitated in ice-cold diethyl ether, and methanol (few drops), next filtered and dried, to give a white powder (yield: ~90%). The PLGA–PEG-DCL or PLGA-PEG-AG copolymers were obtained by adding a solution of DCL salt (128.3 mg, 0.296 mmol, 4 equiv), or AG (77.6 mg, 0.296 mmol, 4 equiv) in DMF (3 mL), respectively, and DIPEA (38.26 mg, 0.296 mmol, 4 equiv) to a solution of PLGA–PEG-NHS (1.5 g, 0.074 mmol) in dimethylformamide (DMF, 8 mL). The reaction mixture was magnetically stirred at room temperature for 24 h, under nitrogen atmosphere. The progress of reaction and the purity of PLGA-PEG-DCL and PLGA-PEG-AG copolymers were determined by high performance liquid chromatography (HPLC) on a Hypersil BDS C18 column (Alltech Italy), (250 × 4.6 mm i.d., 5 μm particle size), using as eluent a linear gradient of eluent B (95% MeCN, 0.07% TFA) in A (0.1% TFA) from 15 to 100% for 30 min (flow-rate 1 mL/min, temperature at 25 °C and a detector wavelength of 280 nm). The equipment consisted of an HP 1200 (Agilent Technologies, USA) system, controlled by HP ChemStation software, including an autosampler and a diode array detector. After purification, a white solid was obtained (yield: ~70%), which was characterized by ^1^H-NMR and UV analysis. For conjugation, we noted that increased equivalent ratio of PLGA-PEG-NHS and DCL or AG (to 1:5) improved the conjugation efficiency. The polymers were then freeze dried and stored at −20 °C before use.

### Preparation of NPs

NPs, prepared by a nanoprecipitation method, are composed of a blend of two polymers, poly(epsilon)-caprolactone (PCL) and PLGA-PEG-A or PLGA-PEG-DCL or PLGA-PEG-AG. Briefly, PCL and PLGA-PEG-ligand conjugated polymer, with a mass ratio of 1.5:1, and EGCG (4%, w/w), were dissolved in acetonitrile and added dropwise under gentle stirring to an aqueous solution of Pluronic F-127 (0.1% w/w) giving a final polymer concentration of 7.0 mg/mL. The resulting suspension was stirred at room temperature to evaporate the organic solvent, then centrifuged and washed to remove the non-encapsulated EGCG. EGCG-free NPs (indicated as A-NPs, DCL-NPs, and AG-NPs, on the base of the respective polymer used for their preparation, i.e. PLGA-PEG-A, PLGA-PEG-DCL, and PLGA-PEG-AG, respectively) were produced in a similar manner and used as comparison. The fluorescent FITC-loaded NPs (FITC-NPs) were prepared, as above mentioned, by adding fluorescein isothiocyanate (FITC) instead of EGCG to different polymer solutions.

### Morphology examinations

The morphology of NPs was examined by Scanning Electron Microscopy (SEM) (model DSM 962; Carl Zeiss Inc., Germany). A drop of NPs aqueous suspension was placed on aluminum stub and dried under vacuum for 12 h. The samples were then analyzed at 20 kV acceleration voltage after gold sputtering, under an argon atmosphere.

### Measurement of particle size and polydispersity index

Particle size (PS) and polydispersity index (PI) were measured using Dynamic Light Scattering (DLS, Zetasizer Nano ZS; Malvern Instruments, UK) at 25 °C, and a fixed angle of 90°. Each sample was suitably diluted and measured in triplicate.

### Measurement of zeta potential

Zeta potential of NPs was measured at 25 °C with a Zetasizer Nano ZS under an electrical field of 40 V/cm. The samples were diluted and sonicated for several minutes before measurement. The data were obtained with the average of three measurements.

### EGCG loading content, encapsulation efficiency and yields of production

The amount of EGCG encapsulated was determined by dissolving an aliquot of NPs weighed accurately (about 1.5 mg in 1 mL of acetonitrile). The solution was filtered through a 0.2 μm syringe filter and analyzed spectrophotometrically (Cary 3, Varian) at 275 nm. The drug loading content (DLC%), drug entrapment efficiency (EE%), and yield of NPs (YP%) were presented by the following equations, respectively:


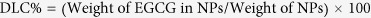



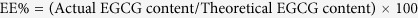



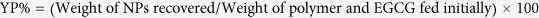


### *In vitro* EGCG release study

The *in vitro* release study of EGCG from NPs was performed by dialysis method. For this purpose, about 1.0 mL of EGCG-loaded NPs and pure EGCG in PBS solution (pH 7.4) was placed into dialysis bag (MWCO: 3.5 KDa), and dialyzed against 20 mL of release medium under gentle stirring at 37 °C. At each time point, 1.0 mL of external release medium was withdrawn and replaced with an equal volume of the fresh medium. Samples were filtered and EGCG concentration was assayed spectrophotometrically at 275 nm. Percent cumulative release of EGCG was plotted v/s time. Each experiment was performed in triplicate.

### Cell culture, treatment, and western blotting

Prostate carcinoma cells LNCaP (hormone-sensitive cells), PC-3 and DU-145 (hormone-independent line possessing dysfunctional androgen receptors), and normal primary prostate epithelial cells (PrEC), were obtained from American Type Culture Collection (Manassas, VA, USA). The cancer cells were cultured in RPMI 1640, the cells were maintained under standard cell culture conditions supplemented with 10% FBS and 1% penicillin/streptomycin at 37 °C and a 5% CO_2_ environment. The normal epithelial cells were grown in epithelial cell media obtained from American Type Culture Collection. The cells (60–70% confluent) were treated with native EGCG or its nanoformulation(s) in complete medium at a dose of 20 μM. Forty-eight h later, the medium was aspirated and the cells were washed with cold PBS (10 mmol/L, pH 7.4) followed by incubation in an ice-cold lysis buffer [50 mmol/L Tris–HCl, 150 mmol/L NaCl, 1 mmol/L EGTA, 1 mmol/L EDTA, 20 mmol/L NaF, 100 mmol/L Na_3_VO_4_, 0.5% NP40, 1% Triton X-100, 1 mmol/L phenylmethylsulfonyl fluoride (pH 7.4)] with a freshly added protease inhibitor cocktail (Protease Inhibitor Cocktail Set III, Calbiochem, La Jolla, CA) over ice for 30 min. The cells were scraped and the suspension was collected in a microfuge tube and passed through a 21-gauge needle to break up any cell aggregates. The lysate was cleared by centrifugation at 14,000 × *g* for 25 min at 4 °C, and the supernatant (total cell lysate) was stored at 80 °C for further analysis. For Western blotting, 25–40 μg of protein were resolved over 8–12% polyacrylamide gels and transferred to a nitrocellulose membrane. The blot was blocked in blocking buffer [7% nonfat dry milk/1% Tween 20; in 20 mmol/L TBS (pH 7.6)] for 1 h at room temperature, incubated with the appropriate monoclonal or polyclonal primary antibody in blocking buffer for 2 h at room temperature or overnight at 4 °C, followed by incubation with an appropriate secondary antibody HRP conjugate. The blots were exposed to enhanced chemiluminescence (Thermo Scientific Pierce, Rockford, IL) and subjected to autoradiography using a BioRad (Hercules, CA) imaging system. Densitometric measurements of the bands in the Western blot analysis were done using the digitalized scientific software program Quantity One (BioRad). The treatment protocol was performed at least three times and the individual protein expressions were analyzed three times with comparable results.

### Cell viability assay

Cell viability was assessed by a 3-[4,5-dimethylthiazol-2-yl]- 2,5-diphenyltetrazoliumbromide (MTT) assay as described previously. Briefly, the cells (1 × 10^4^) were plated per well in 200 μL of complete culture medium. The next day, cells were treated with free EGCG or EGCG-loaded non-targeted or targeted NPs at a dose of 20 μM for 48 h. Each concentration was repeated in 12 wells. After incubation for the specified time at 37 °C in a humidified incubator, cell viability was determined. Media was replaced with MTT (5 mg/mL in PBS) in each well and incubated for 2 h, after which the MTT solution was aspirated from the wells and 0.2 mL buffered DMSO was added. After a 10 min mixing, the absorbance was recorded on a microplate reader at a wavelength of 540 nm. The effect of each agent on growth inhibition was assessed as a percentage of cell viability in which vehicle-treated cells were taken as 100% viable. The experiment was repeated three times with similar outcomes.

### Flow cytometry

Untreated and treated PCa cells (2 × 10^5^) were collected, washed twice with PBS and fixed with 4% formaldehyde for 10 min at room temp, followed by cell permeabilization with 1% Triton-X100. The cells were again washed twice with PBS then blocked with 10% normal goat serum for 15 min at room temperature and incubated with anti-DR4 antibody at dilution recommended by vendor for 4 h at 4 °C. Goat-anti-mouse FITC antibody (1:50) was used as secondary antibody for 30 min in dark. Cells were washed with PBS with 0.5% BSA twice and subjected to flow cytometry. For isotype control 20 μL of the FITC mouse IgG1 was used. After staining, flow cytometric analysis was performed with a LSRII (BD Biosciences, CA) at the UWCCC Flow Cytometry Facility using FlowJo software (Treestar, OR).

### Cellular uptake

Fluorescence microscopy was utilized to examine the cellular uptake of the FITC encapsulated in our novel nanosystems. Cells were plated in a 2-chambered slide at 5000 cells/well and allowed to grow for 18 h. Post attachment, the cells were incubated for 30 or 120 min with FITC encapsulating NPs both in PSMA responsive and unresponsive PCa cells. Unbound NPs were removed by washing three times with cold PBS and the cells were mounted using ProLong^®^ Gold Antifade Reagent (Life Technologies, Grand Island, NY). The mountant was allowed to cure overnight in the dark at room temperature. The images were captured using a camera equipped on a Nikon Eclipse Ti inverted microscope (Nikon Instruments, Inc., Melville, NY). The experiment was repeated four times with similar results.

### *In vivo* tumor xenograft in athymic nude mice

The effects of EGCG and its nanoformulations were studied *in vivo* using athymic nude mice xenograft model. Athymic (nu/nu) male nude mice (Charles River laboratories. Wilmington, MA) were housed under pathogen-free conditions with a 12 h light/12 h dark schedule and fed with an autoclaved diet ad libitum. Forty-eight male athymic nude mice (4 weeks of age) were housed (four per cage) and fed autoclaved diet and water *ad libitum*. 2 million 22 R*v*1 cells (1:1; media: matrigel) were implanted subcutaneously below the shoulders. Mice were randomly distributed into four groups of 12 each. 24 h post cell inoculation, Group I received void NPs or PBS (6 mice each) and served as the control; Group II received EGCG (1 mg/mice/day); Group III received non-targeted A-EGCG NPs (100 mg/mice/day); and Group IV received targeted DCL-EGCG NPs (100 mg/mice/day). All treatments were provided five times a week. The tumor size was measured by determining two perpendicular dimensions with calipers, and the volume was calculated using the formula (axb^2^)/2, where “a” is the longer, and “b” is the smaller dimension.

Throughout the experiment the animals were evaluated for body weight, consumption of food and apparent signs of toxicity. At termination of experiment, phlebotomy was performed to obtain sera for PSA estimation by enzyme-linked immunosorbent assay.

The studies were conducted according to the Institutional guidelines for the care and use of animals and were approved by Animal Care and Use Committee, School of Medicine and Public Health, University of Wisconsin-Madison.

### PSA estimation by ELISA

The human PSA Quantikine ELISA Kit (R&D Systems, Minneapolis, MN) was used for the quantitative determination of PSA levels in serum according to manufacturer’s instructions.

### Statistical Analysis

In all statistical analysis, the significance was set at a probability of P < 0.05. All results were reported as the means ± standard error (SEM). Statistical analysis was performed by Student’s t test for two groups, and one-way ANOVA for multiple groups, followed by Newman-Keuls test if P < 0.05.

## Results and Discussion

### Design of targeted NPs

As a further extension of our study^36^, we planned to use poly(D,L-lactide-co-glycolide) poly(ethylene glycol) carboxylic acid (PLGA-PEG-COOH, namely PLGA-PEG-A), as biocompatible/biodegradable polymer for the preparation of NPs. This starting carboxylate-functionalized di-block-copolymer was utilized for the preparation of the other polymers (i.e., PLGA-PEG-DCL and PLGA-PEG-AG), needed to obtain the targeted NPs. These materials enable the generation of NPs with a hydrophilic surface, which confer to NPs antibiofouling properties to limit macrophage capture, as well as to reduce nonspecific interaction, thus preventing NPs loss to side location^45^,^46^. Moreover, hydrophilic PEG also enhanced solubility NPs in blood, resulting in prolonged circulation time, and increasing accumulation in tumors due to the enhanced permeability and retention (EPR) effect^47^,^48^,^49^. Since the PSMA catalytic site contains two zinc ions on catalytic site, with a funnel-shaped tunnel with a depth of approximately 20 Å, PEGylation can also serve as a spacer to ensure optimal distance between the targeting ligand within the active site and the NP surface.

We also used poly(epsilon-caprolactone) (PCL) mixed with PEGylated polymers in order to optimize the preparation of nanosystems, and to ensure rapid precipitation of batches containing the hydrophilic PLGA-PEG copolymer^50^.

As far as the ligands targeting PSMA are concerned, we first addressed to the pseudomimetic dipeptide *N*-[*N*-[(*S*)-1,3-dicarboxypropyl]carbamoyl]-(*S*)-lysine (DCL)^51^ as a PSMA targeting ligand, belonging from a family of compounds capable to bind PSMA with a similar high affinity and specificity to antibodies and aptamers^52^. Moreover, we also proposed an original targeting ligand, the dipeptide L-Aspartyl-L-Glutamate (AG), with a predicted putative binding affinity toward NAALADase or glutamate carboxypeptidase II. Both small organic molecules were also selected for their chemical stability, for their availability (ie, AG), as well as for their relative low cost of production, especially when produced in scaled-up (for DCL). Based on the structural X-ray information of the PSMA binding site and the abovementioned considerations, we thus envisioned to synthesize the tri-block-co-polymers PLGA-PEG-DCL and PLGA-PEG-AG by conjugating PLGA-PEG-A (with a methylene chain of appropriate length) with the ligands (i.e., DCL and the novel ligand AG) to the NPs surface.

### Docking analysis on PSMA active site

Starting from DCL as a model ligand, we investigated the behavior of DCL-PEG conjugation on binding with PSMA by a computational docking experiments. Thus, preliminary comparative analyses between DLC and PEG-DCL, as well as DCL and AG were also carried out. Most specifically, by using a 3.5-Å solved crystal structure of the PSMA with a glutamate-containing PSMA inhibitor (PDB code 2C6C^44^, for details see the Methods section), a comparative docking study was performed. First, DCL was docked to the PSMA active site and peculiar protein-ligand interactions were analyzed. In the next step, PEG-DCL was docked and the binding modes of DCL and PEG-DCL were compared. Then, preliminary structural analyses to establish crucial contact between DCL and the novel AG on the catalytic site have been carried out.

**Figure 2:**
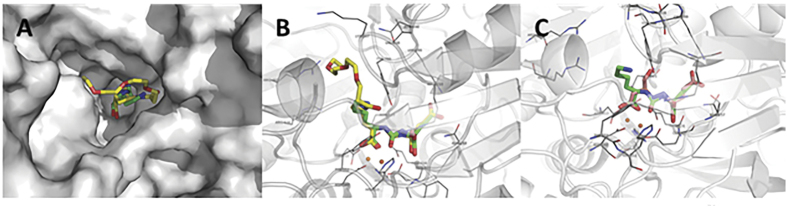
(**A**) PEG-DCL (yellow) and DCL (green) located in the PSMA tunnel; (**B**) binding modes of both PEG-DCL and DCL superimposed on the PSMA active site; (**C**) superimposition of DCL (green) and AG (gray) within the PSMA active site.

MOE software was employed to comparatively dock DCL and the polymer model PEG-DCL (designed with a linker length of >20 Å), as well as DCL and AG, to PSMA. Docking results showed that both DCL and PEG-DCL well accommodated within the active site (Fig. 2A), where the active site metal ions are coordinated by the a DCL carboxylate group and the urea oxygen (Fig. 2B). As previously reported^36^, important interactions are also observed between the carboxylate groups of DCL (and AG), not involved in coordination, with Arg210 and Asn257, and Glu425. PEGylation seem do not affect the interaction of DCL with PSMA active site. Moreover, the superposition of DCL with AG shared a similar disposition on the active, with mutual important interactions in terms of predicted binding affinity (Fig. 2C), thus supporting the use of both ligands for our study.

### Synthesis of di-block-copolymer PLGA-PEG-A

**Figure 3:**
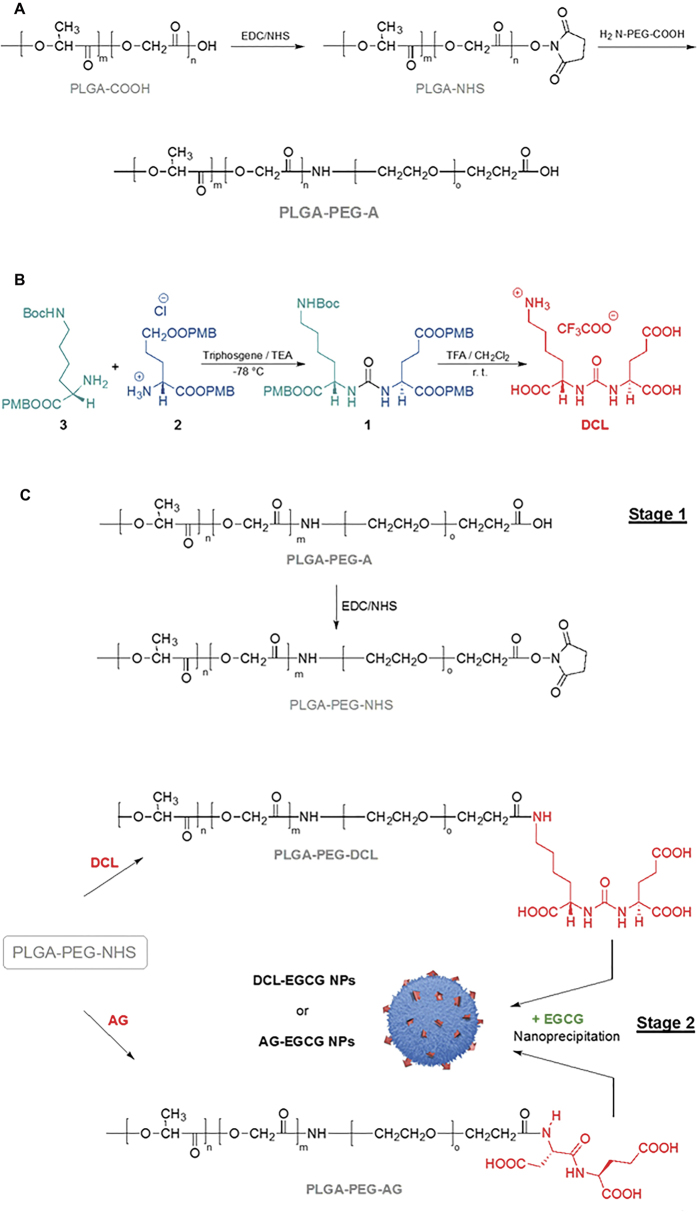
(**A**) Synthesis of copolymer PLGA-PEG-A; (**B**) synthesis of the ligand DCL; (**C**) Synthesis of copolymers PLGA-PEG-DCL and PLGA-PEG-AG (Stage 1), and nanoformulation (Stage 2).

PLGA-PEG-A was prepared by conjugating heterofunctional PEG, NH_2_-PEG–COOH, to PLGA–COOH using standard carbodiimide/NHS-mediated chemistry (Fig. 3A), as previously described^32^,^33^,^36^.

### Synthesis of targeting ligand DCL

The ligand DCL was prepared by following the overall synthetic route previously reported by us^36^, with slight modification, since a scale-up approach was needed to plan *in vivo* assays. Briefly, the key intermediate 2-{3-[1-p-methoxybenzylcarboxylate-(5-tert-butylcarbamylpentyl)]-ureido}-di-p-methoxybenzyl pentanedioate (**1**) was synthesized by coupling of bis-4-methoxybenzyl-L-glutamate hydrochloride (**2**) with triphosgene and TEA at 0 °C, followed by the addition of 2-amino-6-tert-butoxycarbonylamino-hexanoic acid 4-methoxybenzyl ester (**3**) (Fig. 3B). Then, DCL was easily obtained in good yields (~92%) by removing the tert-butyloxycarbonyl (Boc) and the p-methoxybenzyl (PMB) moieties of **1**, using trifluoroacetic acid (TFA)/CH_2_Cl_2_ solution at room temperature. The synthones **2** and **3** were prepared in scale-up following the previously described procedures^36^. DCL and all intermediates were fully characterized by means of NMR, MS and elemental analyses.

### Synthesis of *pseudo*-tri-block-copolymers PLGA-PEG-DCL and PLGA-PEG-AG

The designed PLGA-PEG-DCL and PLGA-PEG-AG *pseudo*-tri-block-copolymers were synthesized by using a two-step reaction, already described for the preparation of the PLGA-PEG-A di-block-copolymer. The carboxyl-capped PLGA-PEG-A was activated with NHS to form the intermediate PLGA-PEG-NHS, which was then converted to PLGA-PEG-DCL or PLGA-PEG-AG after conjugation with the amine group of the DCL or AG (Fig. 3C, stage 1). Characterization of copolymers was performed by ^1^H-NMR and UV spectroscopy.

### Preparation and Characterization of NPs

**Figure 4:**
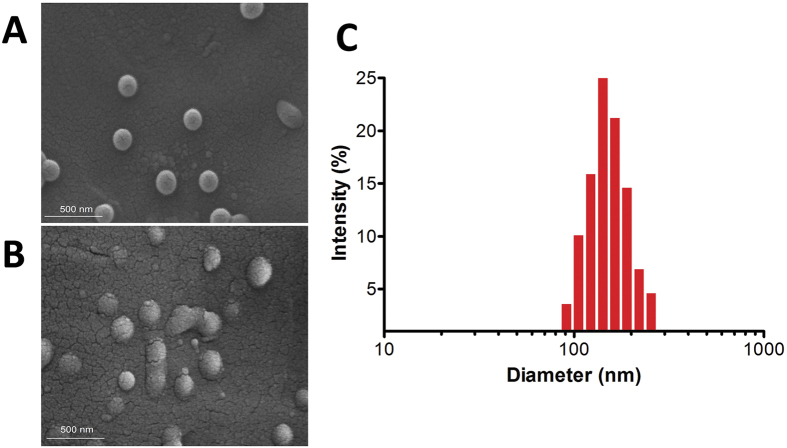
SEM images of A-NPs (**A**), A-EGCG NPs (**B**), and hydrodynamic size distribution of A-NPs (**C**), chosen as examples. The scale bar is 500 nm. The images were collected on 5 different occasions and only representative images are being provided.

The polymeric NPs were formulated using a nanoprecipitation method (Fig. 3C, stage 2), where the hydrophobic PLGA block and PCL self-assemble to form core surrounded by hydrophilic -PEG-(COOH)_n_ chains. Morphology of NPs, investigated by SEM observation, showed well dispersed individual particles with a regular spherical shape (Fig. 4A and Fig. S1, see in Supplementary Information). Due to presence of the EGCG, no difference in the morphological properties of NPs was observed (Fig. 4B).

The hydrodynamic diameter, measured by dynamic light scattering (DLS), and zeta potential of NP batches are summarized in Table 1. All NP samples showed a mean diameter ranging between 130 nm and 250 nm, suitable to avoid reticuloendothelial system uptake and obtain an effective intracellular uptake. In general, the loading of the EGCG during NP preparation determined a considerable increase of size with respect to the corresponding free particles.

In all cases, the NP dispersions exhibit a unimodal particle size distribution, as also confirmed by the obtained low polydispersity indexes (PDI < 0.2) (Table 1), a typical behavior of monodispersed systems, suggesting the formation of homogeneous NP dispersions (Fig. 4C and Supplementary Fig. S2). The zeta potential was negative for both loaded and unloaded NPs as a result of the negatively charged carboxyl groups on the terminal of PLGA-PEG copolymers (Table 1).

As presented in Table 2, the percentage of EGCG content was 1.94%, 2.21% and 1.97%, respectively for batches prepared with PLGA-PEG-A, PLGA-PEG-DCL, and PLGA-PEG-AG, corresponding to 48.5%, 55.3% and 49.3% of encapsulation efficiency. Significant differences were found for the batches based on PLGA-PEG-COOH and PLGA-PEG-DCL polymers. Interestingly, the use of the PCL in the formulation process of NPs resulted in a significant increase of EGCG encapsulation compared to data previously reported^36^. This finding suggest that all three PLGA-PEG-conjugated polymers blended with PCL are able to generate a polymer network characterized by a greatly irregular and disordered crystalline structure which promote a better accommodation of the EGCG molecules. Yields of production ranged between 52% and 75% with an increase of values in the loaded batches with respect to the corresponding EGCG free NPs.

### *In vitro* release kinetics of EGCG from the conjugates

**Figure 5:**
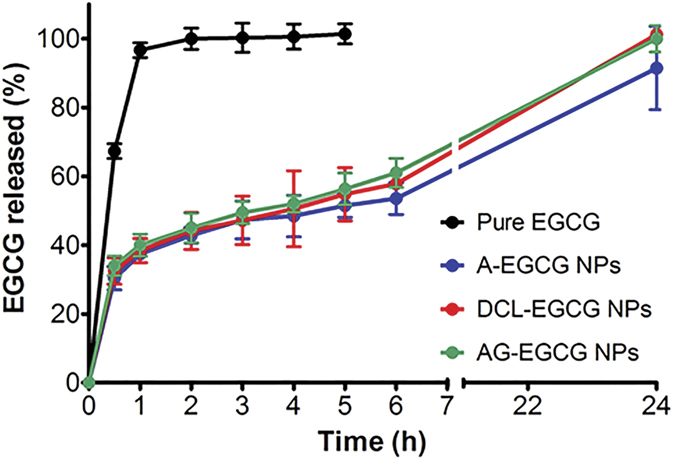
*In vitro* release profiles of EGCG from NPs performed in phosphate buffered saline (pH 7.4) at 37 °C, in comparison with the dissolution rate of the pure EGCG. Data provided is from three experiments conducted separately.

The *in vitro* EGCG release kinetics from non-targeted (A-) and targeted (DCL- and AG-) NPs compared to the dissolution behavior of pure EGCG, are depicted in Fig. 5. The experiments were performed in PBS solution at pH 7.4. Results show that EGCG alone dissolves very quickly (100% within the first hour), being highly soluble in water. Conversely, the encapsulation into NPs determined a control of EGCG release rate with about 50–60% of EGCG released after 6 h that reach 100% in over 24 h. Moreover, amongst the NPs tested, the targeted nanosystems had a slightly better entrapment efficiency with the release happening at a little lower kinetics than the untargeted ones.

### Cytotoxicity of native EGCG and EGCG-loaded NPs

**Figure 6:**
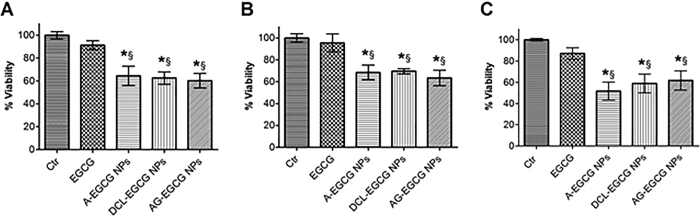
Viability of LNCaP (**A**), PC3 (**B**), and DU-145 (**C**) cells cultured with EGCG-loaded NPs (non-targeted A-EGCG NPs, and targeted DCL-EGCG and AG-EGCG NPs) after 48 h, in comparison with that of pure EGCG at 20 μM dose (n = 3). Each dose was repeated in 6 wells and data from three individual experiments is being used. *Significantly different (P < 0.05) from Control. ^§^Significantly different (P < 0.05) from pure EGCG at equivalent doses.

The effects on the viability of human prostate carcinoma cells, differing in PSMA expression, of EGCG-loaded targeted and non-targeted NPs in comparison to native agent were studied at 48 h post-treatment at a dose of 20 μM. In the tested cells (LNCaP, DU-145, and PC3), we observed a minimal inhibition of cell viability (about 10–15% in all three cell lines) by the native agent (Fig. 6), while all formulations carrying EGCG demonstrated substantial inhibition (about 35–50%) of the viability of all tumor cells. Meanwhile, no significant influence on normal cell viability, using primary prostate epithelial cells (PrEC), was observed for unloaded NPs and A-EGCG NPs, DLC-EGCG NPs, and AG-EGCG NPs, when evaluated at 20 μM, in comparison with the same concentration of EGCG (Supplementary Information Fig. S3).

The observed effects were highly significant (P < 0.05, control or EGCG *vs*. non-targeted and targeted NPs) in LNCaP, DU-145, and PC3 cells, suggesting that this behavior is general and not cell type specific. These results can clearly be attributed to the differential level of EGCG accumulation or cellular uptake, modulated by NPs and/or provided by targeting approach. In fact, the *in vitro* anti-proliferative activity of EGCG encapsulated into the non-targeted NPs (A-EGCG NPs) can be due to a nonspecific cell uptake and to a release of the compound into the medium. On the other hand, the anti-proliferative effect observed after exposure with EGCG targeted NPs can be explained by the binding between NPs and cells, which lead to accumulation and cell uptake. The obtained data for non-targeted and targeted NPs indicate that these mechanisms are balanced and seem to provide similar effect in cytotoxicity.

### *In vitro* uptake by prostate carcinoma cells

**Figure 7:**
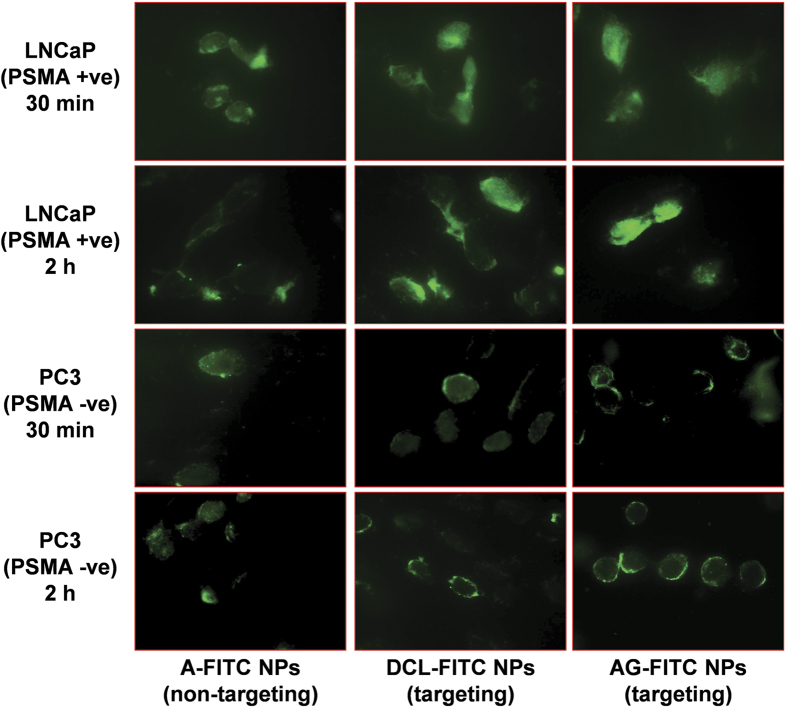
Cellular uptake of the NPs by prostate cancer cells. Panels show representative live cell fluorescence microscopy images of LNCaP (PSMA +ve) and PC3 (PSMA −ve) cells after 30 min and 2 h exposure to non-targeted (A-), and targeted (DCL- and AG-) FITC-loaded NPs. Experiment was repeated 3 times with similar results.

Cellular internalization of EGCG-loaded, non-targeted and targeted NPs was studied in both PSMA expressing (LNCaP) as well as non-expressing (PC3) human prostate carcinoma cells. For the purpose of these experiments we replaced the EGCG with FITC as the encapsulated moiety in the nanosystems. As shown in Fig. 7, the confocal images of prostate carcinoma cells after 30 and 120 min of treatment suggest that the FITC was internalized inside the cells. We observed a clear demarcation of PSMA responsiveness between non-targeted and targeted NPs with LNCaP cells (PSMA +ve) showing localization of the dye in the nucleus, which was very prominent. In PC3 cells, which do not express PSMA, both targeted as well as non-targeted NPs were localized in cytoplasm only. This clearly indicated that targeted EGCG-NPs are selectively internalized by PSMA positive PCa cells.

### Mechanism of action of EGCG-loaded NPs

In the next set of experiments, we tested if the EGCG retains its mechanistic identity even while encapsulated in the nanoformulation. We utilized SDS-PAGE analysis to study the induction of apoptosis and modulation of cell cycle with the different treatments.

**Figure 8:**
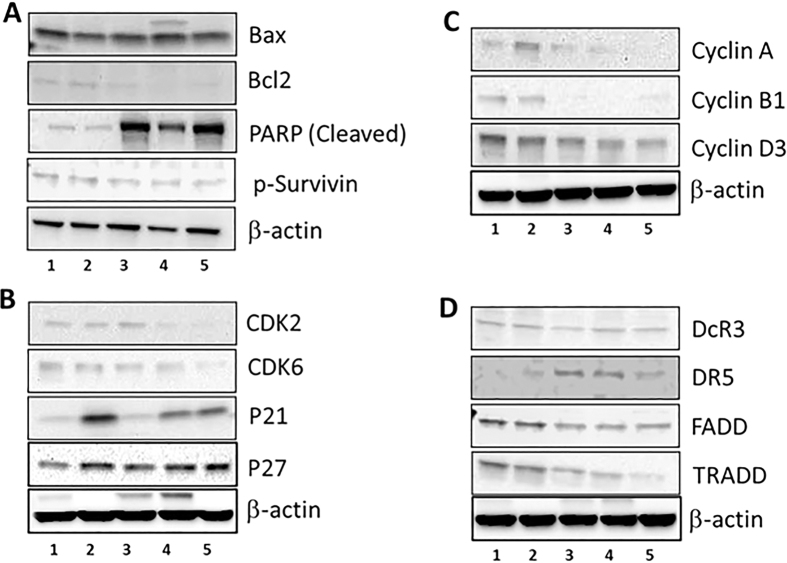
Comparative effects of non-encapsulated EGCG and nano-EGCG on apoptotic biomarkers. (**A**) protein expression of Bax, Bcl2, cleaved PARP, and p-Survivin; (**B**) protein expression of CDK 2, CDK 6, p21 and p27; (**C**) protein expression of cyclin A, B1, and D3; (**D**) protein expression of DcR3, DR3, DR5, FADD, and TRADD. The PC3 cells were treated with each agent and harvested 48 h after treatments. (Lane 1: Control; Lane 2: free EGCG; Lane 3: non-targeted A-EGCG NPs; Lanes 4–5: targeted DCL-EGCG NPs and AG-EGCG NPs. Data is from the experiment repeated twice with similar results, only representative, cropped and unedited images are being provided here.

As expected we did not observe any significant modulation of apoptosis and/or cell cycle regulatory proteins with native EGCG at 20 μM dose. It is widely recognized that the mitochondrial pathway of apoptosis is activated by numerous intracellular and extracellular stress signals, which result in initiation of pro-apoptotic proteins such as Bax and anti-apoptotic proteins such as Bcl-2^19^. Treatment with both non-targeted (A-NPs) and targeted (DCL- and AG-NPs) resulted in an increase in Bax with a concomitant decrease in Bcl-2, thereby shifting the Bax/Bcl-2 ratio in favor of apoptosis (Fig. 8A). In addition, these formulations also resulted in significant cleavage of PARP proteins and inhibition of Survivin (Fig. 8A). We further observed modulation of cell cycle regulatory proteins with the treatments (Fig. 8B and C) suggesting the involvement of this pathway in the observed effects. As shown in the figure, we observed a significant inhibition of cell cycle regulatory proteins (CDK2 and 6; cyclins A, B1 and D3) with all nanoformulations of EGCG in contrast to the native agent. These effects were supplemented with our observations that there was marked induction of the cyclin-dependent kinase inhibitors WAF1/p21 and CIP1/p27, here however the native EGCG was also observed to have comparable effects (Fig. 8B). Lastly, we analyzed the modulation of death-receptor pathway proteins and observed an induction of DR5 protein expression with subsequent inhibition of FADD and TRADD protein expression suggesting an involvement of this pathway in the observed effects (Fig. 8D).

**Figure 9:**
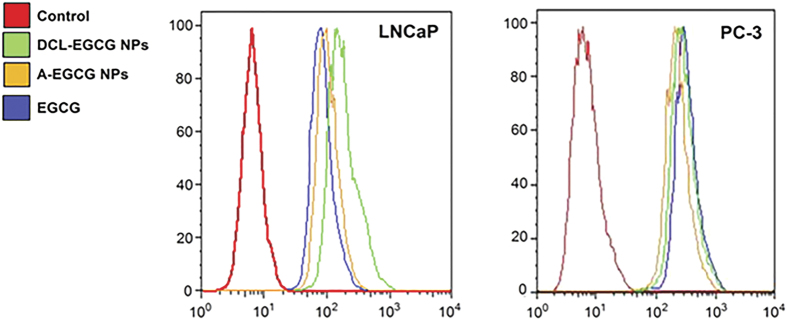
Expression of TRAILR1 in human prostate carcinoma cells PC3. Flow cytometric histograms showing intracellular DR4 staining. Different treatments are shown by different colors. The provided image is representative of two different experiments.

Since DR4 transduces cell death signal and induces cellular apoptosis, we incorporated flow cytometry analysis to look at the expression pattern of this protein post-treatment in PCa cells. As shown in Fig. 9, we observed a clear shift in DR4 staining of both cell types (PSMA +ve and −ve); however targeted nanosystems resulted in a much prominent shift in LNCaP cells (PSMA +ve), in contrast, PSMA negative PC3 cells demonstrated a pattern of similar level of staining amongst all treatments including the native EGCG.

In summary, it seems that EGCG NPs resulted in marked induction apoptosis over native EGCG at the same concentration, and that the concentration required to achieve this modulation was significantly reduced by nanoformulation. Thus, we can reasonably conclude that nanoformulation can have a major impact in induction of apoptosis than free EGGC.

### *In vivo* relevance of the *in vitro* data

**Figure 10:**
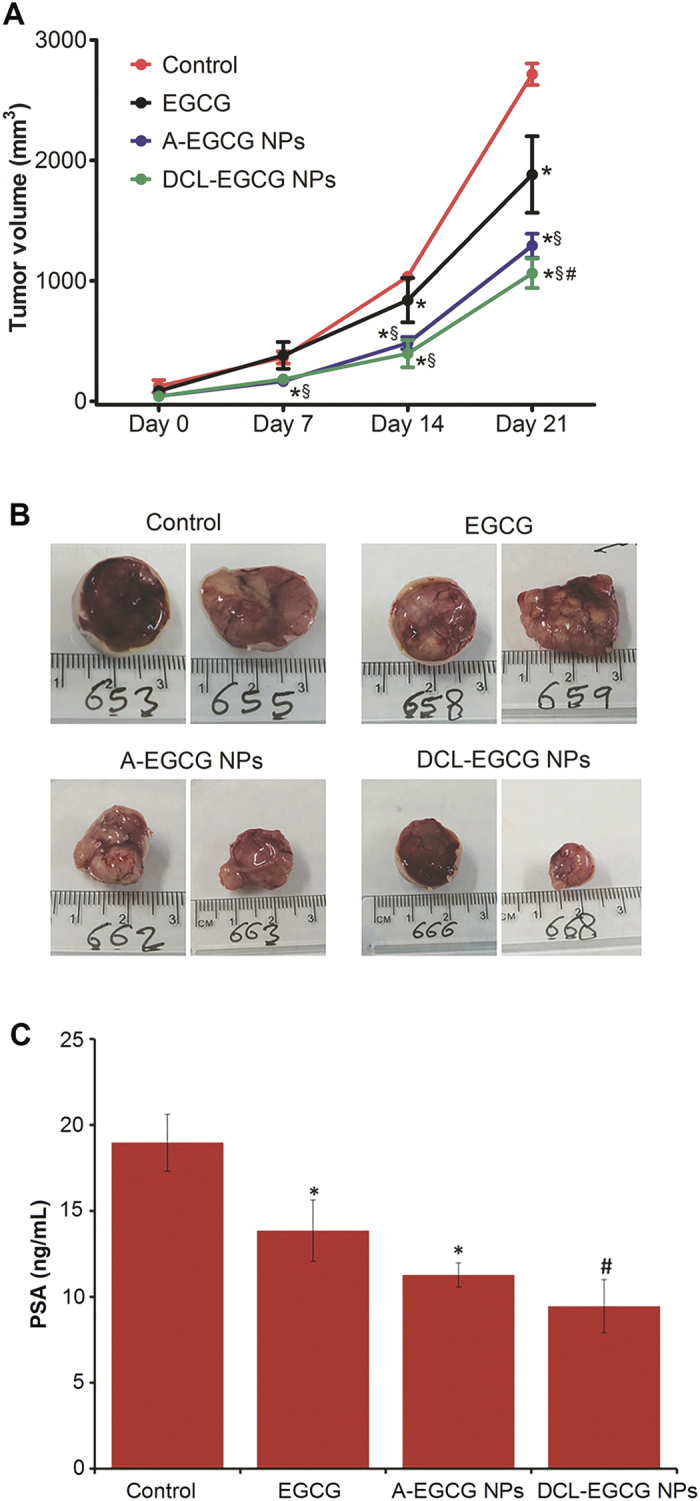
(**A**) Mean volume of tumors after *in vivo* administration of free EGCG, non-targeted (A-EGCG) and targeted (DCL-EGCG) NPs in athymic nude mice xenograft model. The mice were injected subcutaneously with 22 R*v*1 (PSMA +ve) cancer cells, and the treatment was started one day post cell inoculation. The formulations were administered five times a week and tumor volume was measured using a digital caliper. Data represent mean ± SD (n = 12). *P < 0.05 compared with the data from control group at the respective time point. ^§^P < 0.05 compared with the free EGCG group at the respective time point. ^#^P < 0.05 compared with the A-EGCG NPs group at the respective time point. (**B**) Photographs of tumors from control and treated groups. We reported representative pictures from two independent samples of the excised tumors taken at the termination of the experiment. (**C**) The levels of PSA were determined by ELISA assay and expressed as serum (in ng/mL) ±SE of n = 12 mice. *P < 0.05 compared with the vehicle-treated controls; ^#^P < 0.01 compared with the vehicle-treated controls.

Next, we performed tumor xenograft assay to test the effectiveness of DCL-NPs, chosen as the representative model of targeted nanosystems, and non-targeted A-NPs, in comparison to the native EGCG in athymic nude mice. As reported in Fig. 10A we observed a significant difference in the native agent and nanoformulated EGCG starting with the first week post inoculation with about 50% inhibition of tumor volume on day 7. This advantage with the formulation(s) continued for the rest of the study period with nanoformulated EGCG (100 μg/mice/day) showing better efficacy at day 7, 14 and 21 than native EGCG (1 mg/mice/day). At the termination of the experiment, we observed a 30% inhibition in EGCG treated groups while the non-targeted- and targeted-NPs had 55 and 60% tumor growth inhibition, respectively (Control-2685 mm^3^, Avg. tumor wt. 3 g; EGCG-1895 mm^3^, Avg. tumor wt. 2.1 g; A-EGCG NPs- 1230 mm^3^, Avg. tumor wt. 1.3 g; and DCL NPs- 1073 mm^3^, Avg. tumor wt. 1.1 g) (Fig. 10A and B).

This clearly demonstrated the efficacy of both nanosystems over the native agent in inhibiting the growth of tumors in a mouse model of human PCa. Additionally, statistically significant difference was observed for mice treated with DCL-NPs groups compared with A-NPs (P < 0.05), indicating a better antitumor activity with maximum tumor growth inhibition. The passive targeting, i.e. the extravasion from the blood vessels by EPR effect, can play a key role to explain the increased antitumor potency of A-ECGG NPs (and of DCL-EGCG NPs) with respect to native ECGG. The slightly superior activity of the DCL-EGCG NPs can be attributed, in addition to EPR effect, to the avidity of NPs against PSMA, which presumably would promote an active targeting through receptor-mediated endocytosis. More precisely, following the EPR effect, a maximized accumulation of NPs in proximity of cells can lead to improving cell uptake, which would result in increased intracellular concentration of EGCG. Therefore, the contribute between passive and active targeting could explain why targeted NPs work better *in vivo* than *in vitro*.

### Inhibition of PSA secretion in athymic nude mice

Blood was collected through the retro-orbital bleed at the termination of the animal assay. Quantitative sandwich ELISA was used to determine circulating PSA levels in mouse serum secreted by 22Rν1 tumor xenografts. PSA is a serine protease with prostate-specific expression and when it comes to assessing disease progression it is widely accepted as an invaluable tool and a marker in the detection of early PCa. Any agents that could reduce PSA levels is often considered important for clinical implications of PCa. It is currently the most accepted marker for assessment of PCa progression in humans and is being detected in the serum of patients with prostate diseases including prostatitis, benign prostatic hypertrophy and PCa^53^. We observed a significant inhibition of PSA release in the host blood with targeted NPs showing about 50% lower PSA than the control group (Fig. 10C). This data set clearly corroborated our observation of tumor growth inhibition by the different nanotechnology based formulations of EGCG.

## Conclusions

Drug-encapsulated NPs have the potential to improve effectiveness of current cancer chemotherapies, particularly using chemopreventive natural bioactive compounds, by increasing drug efficacy, lowering drug toxicity, and maintaining a relatively high concentration of drug at the site of interest. This is owed to more specific targeting to tumour tissues via improved pharmacokinetics and pharmacodynamics and active intracellular uptake. Following targeted nanotechnology strategy, in this study we developed novel nanosystems encapsulating EGCG, which demonstrated effective antiproliferative activity under *in vitro* conditions and significant increase of tumor growth inhibition in mouse xenograft model experiments, with respect to native compound. We obtained NPs with optimal combination of physicochemical characteristics, which are needed for preclinical evaluation. Importantly, NPs composed of a blend of two polymers, PLGA-PEG-A or PLGA-PEG-DCL, or PLGA-PEG-AG, with PCL, resulted precisely controllable in terms of EGCG loading content, encapsulation efficiency and yields of production, peculiar issues for scale-up production.

We further demonstrated that functionalization of polymeric nanocarriers with some small low molecular weight molecules with high affinity toward the target PSMA antigen present on PCa cells significantly enhanced the anticancer potential of EGCG *in vivo*. Although the EGCG increased its ability to induce apoptosis when loaded into NPs, it seems to behave differently if encapsulated into the non-targeted (A-EGCG) or targeted (DCL-EGCG NPs or AG-EGCG NPs) NPs in tumor growth inhibition. These results could provide a promising and a most effective platform for the development of our nanoprototypes as efficient drug delivery system for chemoprevention/therapy of PCa in clinic.

## Additional Information

**How to cite this article:** Sanna, V. *et al*. Targeted nanoparticles encapsulating (−)-epigallocatechin-3-gallate for prostate cancer prevention and therapy. *Sci. Rep.* **7**, 41573; doi: 10.1038/srep41573 (2017).

**Publisher's note:** Springer Nature remains neutral with regard to jurisdictional claims in published maps and institutional affiliations.

## Supplementary Material

Supplementary Information

## Figures and Tables

**Figure 1:**
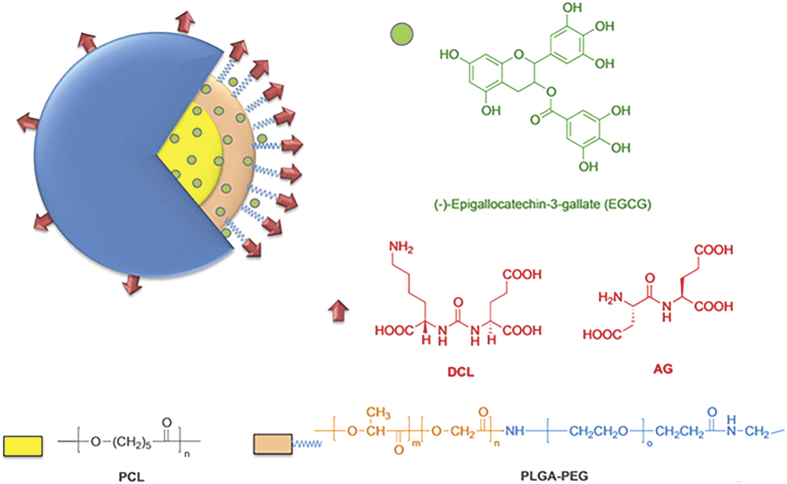
Schematic representation of the designed targeted EGCG NPs. Chemical structure of (−)-epigallocatechin-3-gallate (EGCG), the PEGylated PLGA polymers (PLGA-PEG), and the targeting ligands DCL and AG.

**Table 1:** Mean diameter, polydispersity index (PDI), and zeta potential (ζP) of formulated NPs.

Sample	Mean diameter (nm)	PDI	ζP (mV)
A-NPs	131.13 ± 0.91	0.127 ± 0.010	−21.3 ± 8.35
A-EGCG NPs	150.80 ± 0.69	0.127 ± 0.012	−32.1 ± 8.80
DCL-NPs	174.70 ± 1.65	0.141 ± 0.008	−24.3 ± 8.72
DCL-EGCG NPs	209.13 ± 1.74	0.192 ± 0.015	−30.0 ± 7.08
AG-NPs	134.27 ± 0.32	0.183 ± 0.019	−24.5 ± 8.23
AG-EGCG NPs	251.93 ± 1.25	0.218 ± 0.006	−29.9 ± 9.97

Data are mean ± S.D., n = 3.

**Table 2:** Percentage of Encapsulation efficiency (EE), EGCG loading content (DLC), and yields of production (YP) of formulated NPs.

Sample	%EE	%DLC	%YP
A-NPs	—	—	53.33 ± 2.44
A-EGCG NPs	48.51 ± 2.43	1.94 ± 0.10	74.84 ± 2.53
DCL-NPs	—	—	52.44 ± 3.13
DCL-EGCG NPs	55.31 ± 1.71	2.21 ± 0.07	65.24 ± 3.43
AG-NPs	—	—	53.33 ± 2.44
AG-EGCG NPs	49.26 ± 3.60	1.97 ± 0.14	62.04 ± 2.52

Data are mean ± SD, n = 3.
