# Supplementary material for: Targeted nanoparticles encapsulating (−)-epigallocatechin-3-gallate for prostate cancer prevention and therapy
Source: Sci Rep. 2017 Feb 1;7:41573. doi: 10.1038/srep41573 (PMC5286400; doi:10.1038/srep41573)

Supporting Information

Targeted nanoparticles encapsulating (-)-epigallocatechin-3-gallate for prostate cancer prevention and therapy

Vanna Sanna, Chandra K. Singh, Rahime Jashari, Vaqar M. Adhami, Jean Christopher Chamcheu, Islam Rady, Mario Sechi, Hasan Mukhtar and Imtiaz A. Siddiqui

Figure S1. SEM images of A-NPs (A), A-EGCG NPs (B), DCL-NPs (C), DCL-EGCG NPs (D), AG-NPs (E), and AG-EGCG NPs (F).

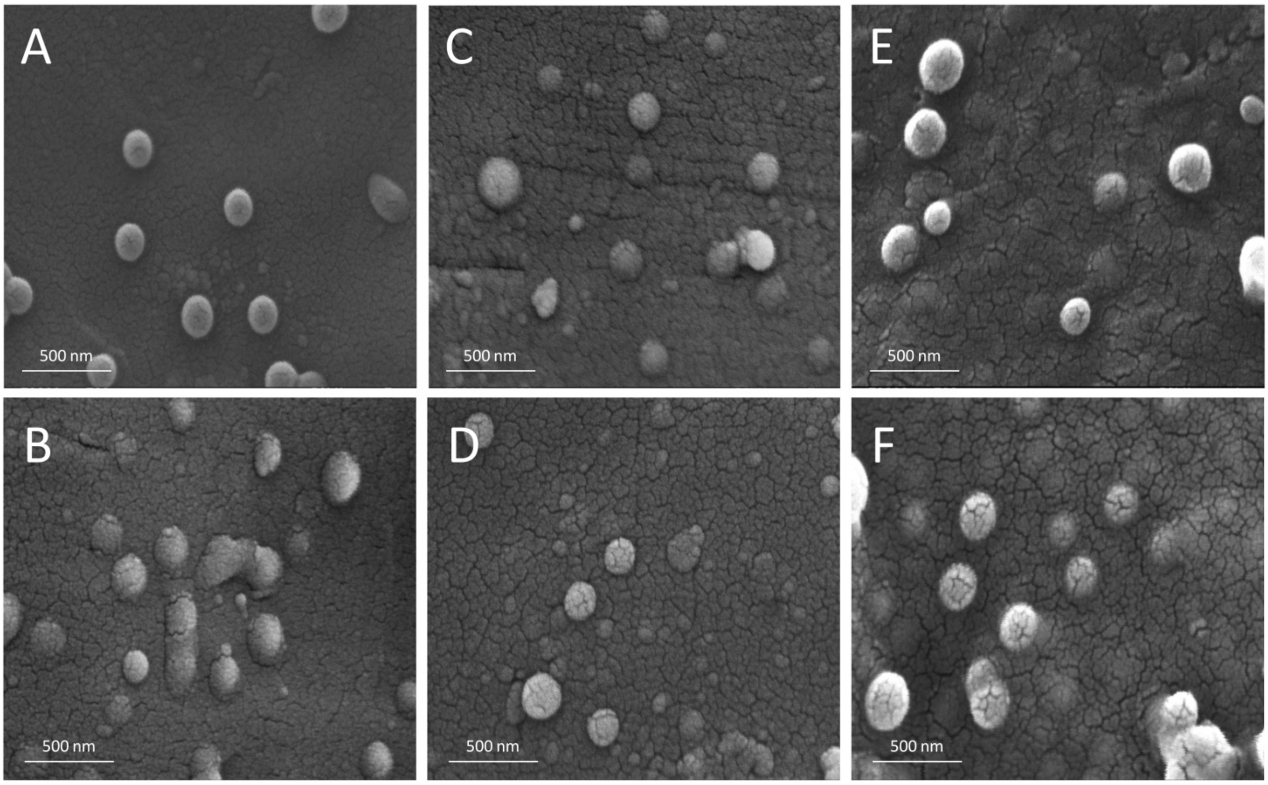

Figure S2. Size distribution of A-NPs (A), A-EGCG NPs (B), DCL-NPs (C), DCL-EGCG NPs (D), AG-NPs (E), and AG-EGCG NPs (F).

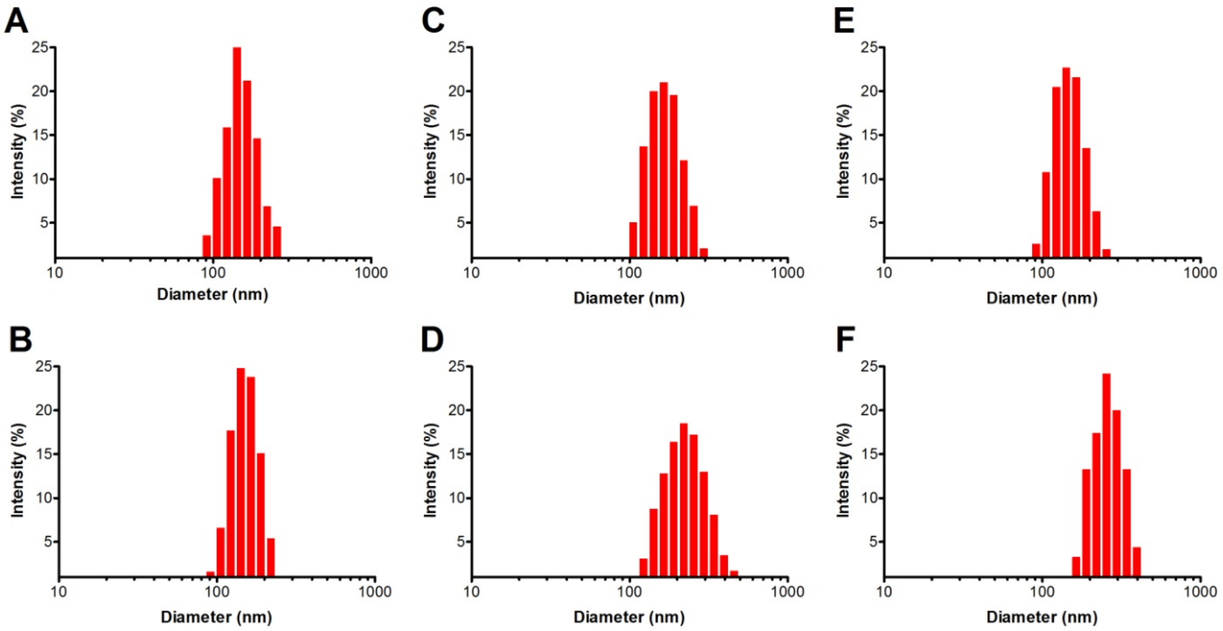

Figure S3. Viability of PrEC cells cultured with unloaded and loaded NPs after 48 h, in comparison with that of pure EGCG at 20 μM dose. Non-encapsulated NPs were run in parallel and used in equal amount to the EGCG-loaded NPs. Each treatment was repeated in 8 wells and data from three individual experiments is being used.

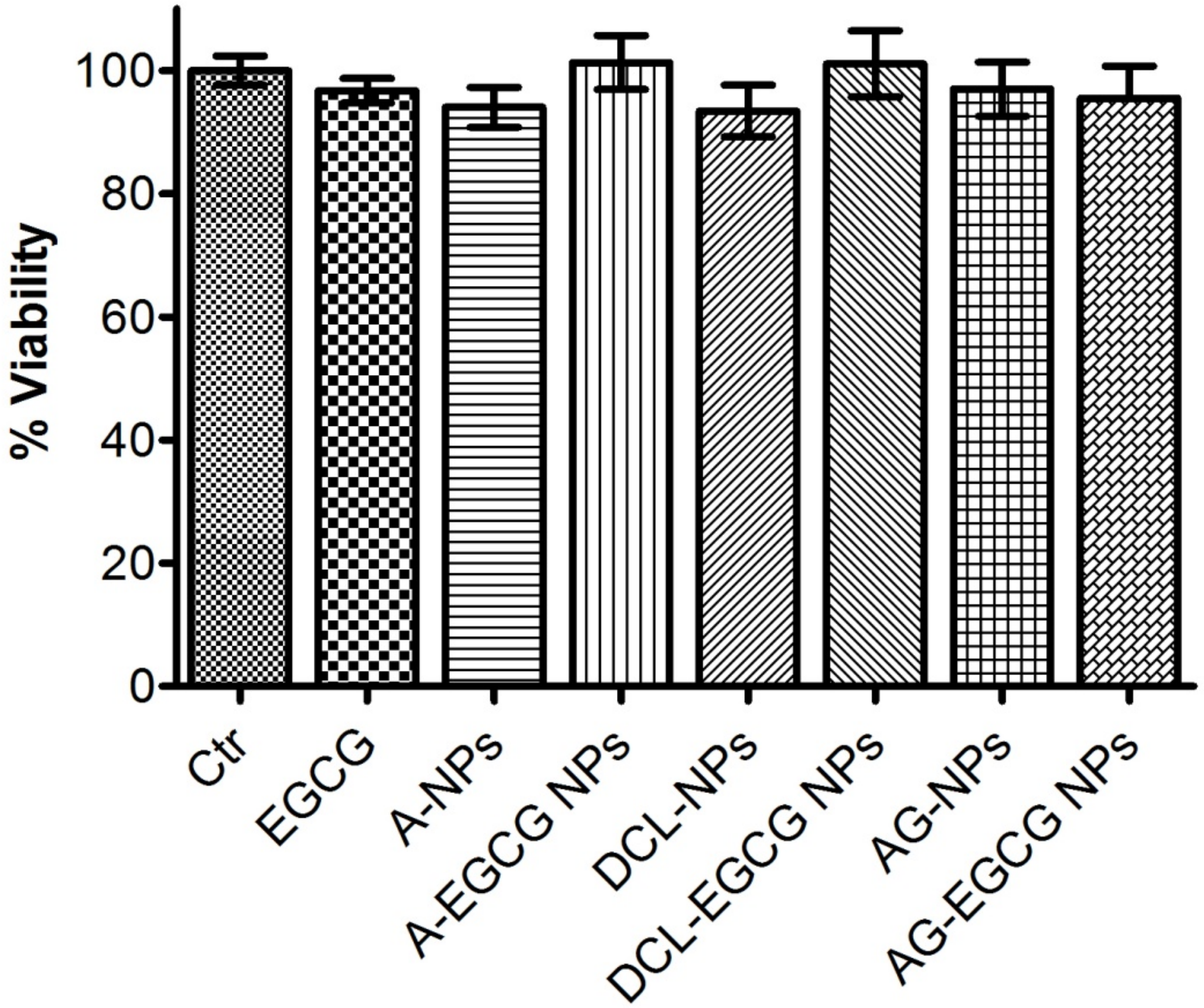

Supplement: Supplementary Information [file srep41573-s1.pdf]
